# Hit-and-run: a Swedish nationwide cohort study of serious transport accidents and convictions due to traffic offenses in obsessive–compulsive disorder

**DOI:** 10.1007/s00127-021-02182-x

**Published:** 2021-11-15

**Authors:** David Mataix-Cols, Lorena Fernández de la Cruz, Gustaf Brander, Erik Andersson, Brian M. D’Onofrio, Christian Rück, Henrik Larsson, Paul Lichtenstein, Anna Sidorchuk

**Affiliations:** 1 Department of Clinical Neuroscience, Child and Adolescent Psychiatry Research Centre, Karolinska Institutet, Gävlegatan 22B, 8th floor, 113 30 Stockholm, Sweden; 2 Stockholm Health Care Services, Region Stockholm, Stockholm, Sweden; 3 Science for Life Laboratory, Department of Medical Biochemistry and Microbiology, Uppsala University, Uppsala, Sweden; 4 Division of Psychology, Department of Clinical Neuroscience, Karolinska Institutet, Stockholm, Sweden; 5 Department of Medical Epidemiology and Biostatistics, Karolinska Institutet, Stockholm, Sweden; 6 Department of Psychological and Brain Sciences, Indiana University, Bloomington, IN USA; 7 School of Medical Sciences, Örebro University, Örebro, Sweden

**Keywords:** Obsessive–compulsive disorder, Accidents, Traffic, Motor vehicles, Injuries, Mortality

## Abstract

**Purpose:**

Individuals with obsessive–compulsive disorder (OCD) often report driving-related obsessions, such as fears of causing accidents, but the risk of transport accidents in OCD is unknown. We investigated whether individuals with OCD have an increased risk of serious transport accidents and convictions due to traffic offenses and explored the role of psychiatric comorbidities.

**Methods:**

We included all individuals ≥ 18 years living in Sweden between 1997 and 2013 (*N* = 5,760,734). A total of 23,126 individuals had a diagnosis of OCD in the National Patient Register. We also identified 16,607 families with full siblings discordant for OCD. Cox proportional hazards regression models estimated hazard ratios (HRs) with 95% confidence intervals (CIs) for the risk of three outcomes in individuals with OCD, compared to unexposed individuals and their unexposed full siblings: injuries or deaths due to transport accidents, injuries or deaths due to motor vehicle accidents, and convictions related to traffic offenses. Psychiatric comorbidities were systematically adjusted for.

**Results:**

Women, but not men, with OCD had a marginally increased risk of serious transport accidents (adjusted HR = 1.20 [95% CI 1.13–1.28]) and motor vehicle accidents (adjusted HR = 1.20 [95% CI 1.09–1.31]), compared to unaffected individuals. Neither women nor men with OCD had a significantly increased risk of convictions. The sibling comparisons showed no significant associations. When psychiatric comorbidities were adjusted for, several observed associations became non-significant or inversed (HRs and 95% CIs below one).

**Conclusion:**

The risks of serious transport accidents and driving-related criminal convictions in OCD are negligible and heavily influenced by psychiatric comorbidity.

**Supplementary Information:**

The online version contains supplementary material available at 10.1007/s00127-021-02182-x.

## Introduction

Fears of harming oneself or others and pathological doubt about whether harm has been caused are amongst the most common symptoms of obsessive–compulsive disorder (OCD) [1–3]. Patients often report fears associated with driving vehicles, such as fears of running over pedestrians or being ‘careless’ and responsible for causing traffic accidents. These symptoms can be very debilitating and are sometimes referred to as ‘hit-and-run’ OCD [4]. Obsessions and doubt are nearly always associated with extensive avoidance behaviour (e.g., avoiding certain roads; not driving at all), compulsive checking (e.g., rear-view mirror checking; ‘is the hand brake on?’), and reassurance seeking (e.g., ‘did I run over that person?’; ‘what if that bump on the road was a body under the wheels?’; ‘how do I know that I did not hit somebody with the car?’). This constellation of symptoms may suggest that individuals with OCD are probably cautious drivers and might actually be less likely to be involved in transport accidents or be convicted due to traffic offenses, compared to individuals without OCD.

On the other hand, there are several factors that may increase the risk of transport accidents in persons with OCD. Performing checking rituals while on the road (e.g., turning back to check if somebody has been run over) may lead to distraction, one of the main causes of traffic accidents [5]. Most individuals with OCD have psychiatric comorbidities, some of which are known to be strongly associated with increased risk of transport accidents, such as attention-deficit/hyperactivity disorder (ADHD) [6, 7]. However, in the absence of published literature on the topic, whether OCD is associated with an increased, similar or even decreased risk of transport accidents is unknown. If individuals with OCD had similar or lower risks of transport accidents, compared with the general population, it would provide useful information for licensing authorities, clinicians, and the patients themselves, who would benefit from having access to objective data.

The main aim of this population-based study was to investigate whether individuals with OCD have an increased risk of transport accidents resulting in injuries or deaths and an increased risk of convictions due to traffic offenses, compared to individuals from the general population and their unaffected full siblings. The sibling design allows for a stricter control of potential unmeasured confounders, such as shared familial factors (e.g., genetic factors, socioeconomic status of the family or parental education), which may influence both OCD and the risk of transport accidents. The second aim was to establish the extent to which the association between OCD and transport accidents or convictions, if found, is explained by coexisting psychiatric comorbidity. In particular, we were interested in ADHD, which is frequently comorbid with OCD (around 19% of OCD cases have also ADHD) [8] and strongly associated with transport accidents [6, 7].

We hypothesized that individuals with OCD would have a similar risk of transport accidents, as well as a similar risk of convictions due to traffic offenses, compared to individuals from the general population. We further hypothesized that a subset of individuals with OCD and comorbid ADHD would have significantly higher risks, compared to individuals from the general population.

## Method

### Data sources

This population-based cohort study linked data from a number of nationwide registers in Sweden, by means of the unique personal identification numbers assigned to all Swedish residents at birth or immigration [9]. The linked registers included: (1) the *Total Population Register*, containing information on demographic variables (e.g., sex, birth date) and migration data for all Swedish residents since 1968 [10]; (2) the *National Patient Register* (NPR), which includes information on diagnoses, based on the Swedish version of the International Classification of Diseases (ICD), given in inpatient care settings (from 1964 and from 1973 for somatic and psychiatric data, respectively, with full coverage from 1987) and specialist outpatient services (from 2001) [11]; (3) the *National Crime Register*, including all convictions in Swedish district courts since 1973 [12]; (4) the *Cause of Death Register*, which covers all dates and causes of death, based on ICD codes, of Swedish residents since 1952 [13]; (6) the *Prescribed Drug Register*, which records all prescribed drugs since July 2005 [14]; and (7) the *Multi-Generation Register*, which contains information on the parents of all individuals born in Sweden from 1932 or ever registered in the country since 1961 [15].

### Study population

Figur 1 shows the numbers of included and excluded individuals. The study population included all individuals born in Sweden before 1995 who were living in the country anytime between 1997 (introduction of the tenth revision of the ICD manual [ICD-10] in Sweden) and 2013 (end of the study period) with available information on their biological mother (*n* = 6,335,789). We excluded individuals that had died or emigrated prior to 1997 or before age 18 (*n* = 425,555). Additionally, we excluded those individuals ever diagnosed with epilepsy (ICD-10 codes G40-G41), organic brain disorder (F00-F09) or mental retardation (F70-F79) (*n* = 149,500) since these conditions are known to interfere with driving [16, 17]. The final cohort included 5,760,734 individuals followed up from January 1, 1997 or their 18^th^ birthday (because in Sweden a driver’s license can only be obtained from age 18), whichever occurred last, until the date of any of the outcomes of interest (see below), emigration, death or the end of the follow-up (December 31, 2013), whichever occurred first.

**Fig. 1:**
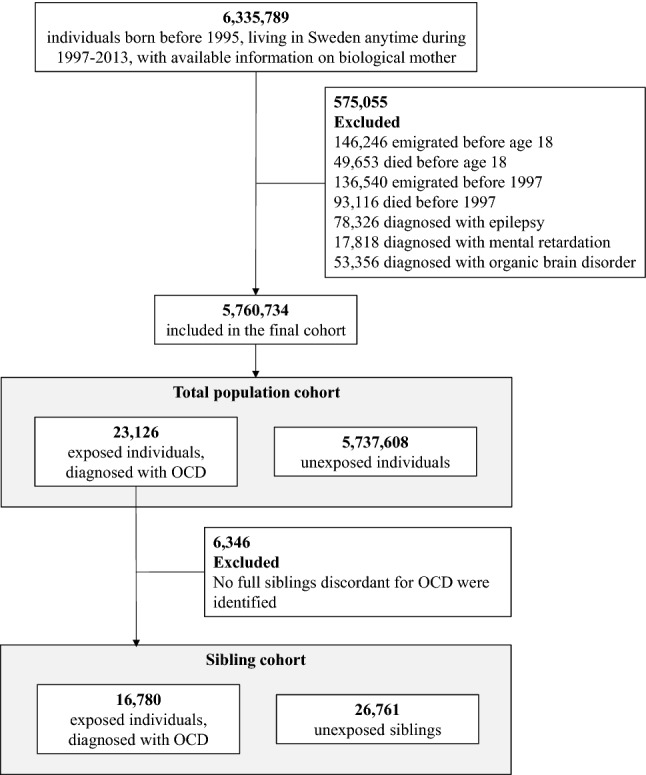
Study population. *OCD* obsessive-compulsive disorder

### Measures

Individuals with an ICD-10 diagnosis of OCD (F42)—registered at age ≥ 6 to avoid diagnostic misclassification [18]—during the study period (i.e., 1997–2013) were collected from the NPR. The ICD-10 codes for OCD in the Swedish NPR have excellent validity and reliability [19]. A ‘lifetime diagnosis’ refers to the diagnosis of OCD recorded in the NPR any time between 1997 and the end of the study period. This is because OCD is an early onset disorder [1] but substantial delays in help-seeking or diagnosis are characteristic of the disorder [20–22]; hence, the recorded date of first diagnosis in the NPR is a poor reflection of the disorder’s actual date of onset. We therefore assume that individuals with a diagnosis of OCD were exposed from the start of follow-up, irrespective of the actual date of diagnosis.

We focused on three related outcomes: records of injuries or deaths due to transport accidents in general, records of injuries or deaths specifically due to motor vehicle accidents, and convictions related to traffic offenses. Records of diagnoses and dates of injuries or deaths due to transport accidents (ICD-10 codes V01-V99) were collected from the NPR and the Cause of Death Register, respectively, if recorded at the age of 18 years or above. These measures refer to a broad group of accidents, including those related to motor vehicles and other transport accidents (e.g., pedestrians or cyclists injured in collision with vehicles). Further analyses specifically focused on the subgroup of motor vehicle accidents (ICD-10 codes V20-29 [motorcycle rider injured in transport accident], V30-V39 [occupant of three-wheeled motor vehicle injured in transport accident], V40-V49 [car occupant injured in transport accident], V50-V59 [occupant of pick-up truck or van injured in transport accident], and V60-V69 [occupant of heavy transport vehicle injured in transport accident]). Additionally, from the National Crime Register, we collected all cases of convictions related to traffic offenses (i.e., reckless driving, hit-and-run offenses, causing death or injury by driving, and moving violations) and the dates of perpetrating the corresponding traffic offenses [23].

From the NPR, we also obtained data on diagnoses of psychiatric disorders recorded in 1997–2013 in order to explore their potential impact on the association between OCD and outcomes of interest. The corresponding ICD-10 codes were retrieved for psychiatric disorders, which were grouped into: (1) autism spectrum disorders (ASD), (2) ADHD (complemented with data from the Prescribed Drug Register on medications used in Sweden for the treatment of this disorder) [24], (3) conduct disorder, (4) anxiety disorders, (5) post-traumatic and other stress-related disorders, (6) eating disorders, (7) depression and other mood disorders, (8) bipolar disorders, (9) schizophrenia and other psychotic disorders, (10) substance use disorders, and (11) dissocial personality disorder (see Supplementary Table 1).

Information on sex and birth year (categorized by 10-year increments) was collected from the Total Population Register.

### Statistical analysis

We conducted five planned analyses. First, we used Cox proportional hazards regression models to estimate hazard ratios (HRs) with 95% confidence intervals (CIs) for the risk of transport accident injuries or deaths and, separately, for the risk of convictions due to traffic offenses, in individuals with ICD-10 diagnoses of OCD, compared to unexposed individuals, with age in years as the underlying time-scale. For injuries and deaths, the analyses included, first, all transport accidents (ICD-10 codes: V01-V99) and, in a subsequent analysis, only the subgroup of motor vehicle accidents (ICD-10 codes: V20-V69). These analyses included the full study cohort of 5,760,734 individuals and all models were initially adjusted for sex and birth year. Tests for interaction indicated that sex modified the association of OCD with two out of three outcomes (for all transport accidents: *p*-value for heterogeneity < 0.001; for motor vehicle accidents: *p* = 0.001; for convictions: *p* = 0.585). Therefore, all analyses were also stratified by sex. To account for effect modification by sex on the association of OCD with transport accidents and motor vehicle accidents (but not convictions), we incorporated an interaction term (OCD × sex) to sex-and birth year-adjusted models for the analysis of the whole cohort.

Second, to reduce the potential bias due to the lack of data coverage (e.g., if the outcome occurred before the start of the follow-up), the main analyses were repeated in a sub-cohort of individuals born from 1979 (i.e., aged ≤ 18 years in 1997) with follow-up available from age 18. This analysis ensures capturing all incident outcome cases and prevents non-differential misclassification of outcome (i.e., if an individual who reached 18 years prior to 1997 had an outcome of interest before the start of follow-up, but appeared to be event-free during the study period).

Third, to address the potential issue of immortal time bias, we excluded individuals with a first recorded OCD diagnosis after age 18 from the sub-cohort born from 1979, and compared the remaining individuals with OCD to the unaffected population. This addresses the concern that individuals with a first OCD diagnosis after age 18 will contribute survival time without being able to have the outcome of or being censored by death before the registered diagnosis, a time period where they statistically are considered ‘immortal’.

Fourth, for the sibling analyses, we identified all individuals with a diagnosis of OCD with information on both parents available from the Multi-Generation Register and then linked them to their full siblings (i.e., those sharing both parents). Family identification numbers were created for the linkage. By design, this comparison controls for potential familial confounders shared by full siblings, including unmeasured shared environmental factors (e.g., parental socioeconomic status, parental psychopathology) and about 50% of the genetic factors. We used stratified Cox proportional hazards regression models where each family was considered a stratum and all full siblings within a family were compared with each other in a way that unexposed siblings served as controls to the exposed ones. These models also initially adjusted for sex and birth year and, as above, an OCD × sex interaction term was then incorporated for the analyses of transport accidents and motor vehicle accidents.

Fifth, to explore the role of psychiatric comorbidities, we repeated the main analysis by adjusting the models for different groups of psychiatric disorders. While the comorbidities with onset in early childhood (e.g., ASD, ADHD, conduct disorder) can be assumed to confound the associations of interest, the role of other comorbidities is less clear as some of them may act as mediators rather than confounders. To avoid over-adjustment for comorbidities with a potential mediating effect, we adjusted for one group of comorbid disorders at a time.

All models were clustered by the identification number of the individual’s mother (for the analyses of the whole study population) or stratified by the family identification number (for the sibling comparison) and used a robust sandwich estimator of standard errors to account for non-independence of observations within families [25]. All tests employed two-tailed significance set at *p* < 0.05. Data management and analyses were performed using SAS, version 9.4 (SAS Institute Inc.) and STATA version 15.1 (StataCorp LLC, College Station, TX, USA), respectively.

## Results

### Descriptive statistics

Table 1 presents the characteristics of 5,760,734 members of the final study cohort (51.09% men; age range 18–65 at the start of follow-up), of which 23,126 individuals (41.66% men) had a diagnosis of OCD recorded in the NPR between 1997 and 2013. Among 9,635 men and 13,491 women with an OCD diagnosis, 79.38% and 82.99% individuals, respectively, had records of other psychiatric comorbidities, with anxiety disorders and depression and other mood disorders being the most common comorbidities.

**Table 1:** Sample characteristics of individuals with obsessive–compulsive disorder and of unaffected individuals from the general population, stratified by sex

	All, *n* (%)		Men, *n* (%)		Women, *n* (%)	
	With OCD	Unaffected	With OCD	Unaffected	With OCD	Unaffected
Individuals at the start of the follow-up	23,126 (100)	5,737,608 (100)	9635 (41.66)^a^	2,933,466 (51.13)^b^	13,491 (58.34)^a^	2,804,142 (48.87)^b^
Age at the start of the follow-up, years^c^						
18–19	12,902 (55.79)	1,713,540 (29.87)	5107 (53.0)	881,119 (30.04)	7795 (57.78)	832,421 (29.69)
20–29	4537 (19.62)	986,547 (17.19)	1870 (19.41)	509,143 (17.36)	2667 (19.77)	477,404 (17.02)
30–39	2848 (12.32)	973,368 (16.96)	1338 (13.89)	498,905 (17.01)	1510 (11.19)	474,463 (16.92)
40–49	1788 (7.73)	970,060 (16.91)	866 (8.99)	494,554 (16.86)	922 (6.83)	475,506 (16.96)
50–65	1051 (4.54)	1,094,093 (19.07)	454 (4.71)	549,745 (18.74)	597 (4.43)	544,348 (19.41)
Psychiatric comorbidities						
Any comorbidity (at least one)	18,844 (81.48)	640,305 (11.16)	7648 (79.38)	303,960 (10.36)	11,196 (82.99)	336,345 (11.99)
Autism spectrum disorders	2367 (10.24)	20,002 (0.35)	1420 (14.74)	12,984 (0.44)	947 (7.02)	7018 (0.25)
Attention-deficit/hyperactivity disorder	3267 (14.13)	64,407 (1.12)	1485 (15.41)	37,618 (1.28)	1782 (13.21)	26,789 (0.96)
Conduct disorder	213 (0.92)	3845 (0.07)	100 (1.04)	2381 (0.08)	113 (0.84)	1464 (0.05)
Anxiety disorders	13,850 (59.89)	308,306 (5.37)	5.179 (53.75)	119,863 (4.09)	8671 (64.27)	188,443 (6.72)
Posttraumatic and other stress-related disorders	4361 (18.86)	132,628 (2.31)	1414 (14.68)	49,162 (1.68)	2947 (21.84)	83,466 (2.98)
Eating disorders	1991 (8.61)	22,589 (0.39)	153 (1.59)	1293 (0.04)	1838 (13.62)	21,296 (0.76)
Depression and other mood disorders	11,186 (48.37)	264,953 (4.62)	4124 (42.80)	104,801 (3.57)	7062 (52.35)	160,152 (5.71)
Bipolar disorders	2395 (10.36)	45,227 (0.79)	778 (8.07)	17,745 (0.60)	1617 (11.99)	27,482 (0.98)
Schizophrenia and other psychotic disorders	2183 (9.44)	44,989 (0.78)	1193 (12.38)	24,321 (0.83)	990 (7.34)	20,668 (0.74)
Substance use disorders	4113 (17.79)	218,021 (3.80)	1951 (20.25)	138,957 (4.74)	2162 (16.03)	79,064 (2.82)
Dissocial personality disorder	112 (0.48)	2078 (0.04)	82 (0.85)	1755 (0.06)	30 (0.22)	323 (0.01)

^a^Percentage of men and women with OCD add up to 100% of 23,126 individuals with OCD

^b^Percentage of unaffected men and women individuals add up to 100% of 5,737,608 individuals from the general population

^c^Individuals are followed from their 18th birthday or January 1, 1997, whichever occurred last

### Risk of serious transport accidents and convictions due to traffic offences

In total, 324,544 individuals had at least one record of injury or death due to a transport accident during the follow-up period (i.e., 1997–2013; mean follow-up: 13.78 years [standard deviation (SD): 5.08]). Among individuals with OCD, 6.86% were involved in serious transport accidents, compared to 5.63% in unaffected individuals, corresponding to no increased risk of this outcome (HR adjusted for sex and birth year [aHR] with an interaction term = 0.99 [95% CI 0.91–1.06]; Table 2). In analyses stratified by sex, men with OCD did not have an increased risk of transport accidents (aHR = 0.98 [95% CI 0.91–1.06]), whereas we observed a slight increased risk in women with OCD, compared to their unaffected counterparts (aHR = 1.20 [95% CI 1.13–1.28]; Table 2).

**Table 2:** Hazard ratios and corresponding 95% confidence intervals for the risk of injury or death due to transport accidents, motor vehicle accidents, and convictions due to traffic offenses among individuals with obsessive–compulsive disorder, compared to unaffected individuals from the general population (population cohort) and their unaffected siblings (sibling cohort)

Population cohort	Individuals with OCD (*n* = 23,126)		Unaffected individuals (*n* = 5,737,608)		HR (95% CI)
	*n*	%	*n*	%	
All serious transport accidents	1587	6.86	322,957	5.63	0.99 (0.91–1.06)^a^
Men	656	6.81	180,239	6.14	0.98 (0.91–1.06)^b^
Women	931	6.90	142,718	5.09	**1.20 (1.13–1.28)**^**‡b**^
Motor vehicle accidents	888	3.84	178,770	3.12	0.94 (0.85–1.03)^a^
Men	418	4.34	111,910	3.81	0.92 (0.84–1.02)^b^
Women	470	3.48	66,860	2.38	**1.20 (1.09–1.31)**^**‡b**^
Convictions due to traffic offenses	97	0.42	20,720	0.36	1.11 (0.91–1.35)^c^
Men	77	0.80	17,376	0.59	1.10 (0.88–1.38)^b^
Women	20	0.15	3344	0.12	1.26 (0.81–1.96)^b^

Sibling cohort	Individuals with OCD (*n* = 16,780)		Unaffected full siblings of individuals with OCD (*n* = 26,761)		HR (95% CI)
	*n*	%	*n*	%	
All serious transport accidents	1167	6.95	1719	6.42	0.96 (0.86–1.09)^a^
Motor vehicle accidents	646	3.85	997	3.73	0.91 (0.78–1.05)^a^
Convictions due to traffic offenses	62	0.37	112	0.42	0.97 (0.69–1.37)^c^

Statistically significant hazard ratios are highlighted in bold. Mean length of follow-up and SD for analyses of outcomes: all transport accidents 13.77 years (SD = 5.08), motor vehicle accidents 13.93 years (SD = 4.99), convictions due to traffic offences 14.11 years (SD = 4.88)

*CI* confidence interval, *HR* hazard ratio, *OCD* obsessive–compulsive disorder, *SD* standard deviation

^a^Adjusted for sex, birth year and interaction term (OCD × sex)

^b^Adjusted for birth year

^c^Adjusted for sex and birth year (interaction term is not included because sex does not modify an association between OCD and convictions)

^‡^*p*-value < 0.001

Injuries or death specifically due to motor vehicle accidents were reported for 179,658 study participants, with 3.84% of OCD individuals and 3.12% of unaffected individuals having a record of motor vehicle accident under the study period (mean follow-up: 13.93 years [SD: 4.99]). Similar to the results seen for all transport accidents, women with OCD diagnoses had a slight increase in risk of motor vehicle accidents (aHR = 1.20 [95% CI 1.09–1.31]), compared to unaffected women, whereas no significant associations were observed among men (aHR = 0.92 [95% CI 0.84–1.02]) or both sexes combined (aHR with interaction term = 0.94 [95% CI 0.85–1.03]; Table 2).

During the follow-up, 20,817 cohort members had at least one recorded criminal conviction due to traffic offences (mean follow-up: 14.11 years [SD: 4.88]). The proportions of individuals with OCD and individuals from the general population with this outcome (0.42% and 0.36%, respectively) were not statistically different in either men or women (Table 2).

In the analysis of the sub-cohort limited to those followed from age 18, the risks for all outcomes attenuated slightly, resulting in weaker but still statistically significant associations of OCD with an increased risk of serious transport accidents and motor vehicle accidents among women (Supplementary Table 2). For the whole sub-cohort and for men, an inverse association of OCD with the risk of transport accidents and motor vehicle accidents was noted. The results for the risk of convictions due to traffic offences showed no significant association but should be interpreted with caution due to limited statistical power (*n* = 32 convictions in the OCD cohort). In the same sub-cohort, when we attempted to control for immortal time bias and compared individuals with OCD diagnoses at age ≤ 18 with their counterparts with no OCD diagnoses under the study, the HRs were further attenuated resulting in no significant associations for OCD and the risk of serious transport accidents and motor vehicle accidents in women (aHR = 1.08 [95% CI 0.85–1.38] and aHR = 1.08 [95% CI 0.77–1.51], respectively), and an inverse association for men (aHR = 0.67 [95% CI 0.49–0.92] and aHR = 0.52 [95% CI 0.34–0.79], respectively). The corresponding analysis for convictions due to traffic accidents was not feasible due to lack of statistical power.

### Sibling comparison

In the study cohort, 1,772,146 families had at least two singleton children*,* of which 16,607 families included clusters of full siblings that were discordant for OCD, totalling 16,780 individuals with OCD and 26,761 unaffected siblings*.* The results of the sibling analysis were comparable to those in the main analysis for the whole cohort, indicating no significant associations between OCD and any of the outcomes of interest (Table 2).

### Role of psychiatric comorbidities

Adjustment for psychiatric comorbidities attenuated all previously obtained HRs, indicating that the observed association of OCD with the outcomes of interest could be better explained by coexisting psychiatric disorders rather than by OCD per se (Table 3). That was, in particular, true for ADHD (as hypothesised), but also for anxiety disorders, post-traumatic and other stress-related disorders, depression and other mood disorders, and substance use disorders. Many of the estimates even became inverse (aHRs and 95% CIs below one), suggesting that the risks in individuals with OCD could be even lower than those among the general population.

**Table 3:** Hazard ratios and corresponding 95% confidence intervals for the risk of injuries and death due to transport accidents, motor vehicle accidents, and convictions due the traffic offenses in individuals with obsessive–compulsive disorder, compared to unaffected individuals from the general population, when adjusting for common psychiatric disorders (one disorder group at a time)

Comorbidities adjusted for	HR (95% CI)								
	All transport accidents			Motor vehicle accidents			Convictions due to traffic offenses		
	All^a^	Men^b^	Women^b^	All^a^	Men^b^	Women^b^	All^c^	Men^b^	Women^b^
Original results	0.98 (0.91–1.06)	0.98 (0.91–1.06)	**1.20 (1.13–1.28)**	0.94 (0.85–1.03)	0.92 (0.84–1.02)	**1.20 (1.09–1.31)**	1.11 (0.91–1.35)	1.10 (0.88–1.38)	1.26 (0.81–1.96)
Attention-deficit/hyperactivity disorder	***0.87 (0.80–0.94)***	***0.85 (0.79–0.92)***	**1.10 (1.03–1.18)**	***0.81 (0.73–0.89)***	***0.79 (0.71–0.87)***	1.08 (0.99–1.19)	***0.64 (0.52–0.79)***	***0.63 (0.50–0.79)***	0.80 (0.51–1.26)
Autism spectrum disorders	0.99 (0.91–1.07**)**	0.99 (0.92–1.07)	**1.19 (1.12–1.27)**	0.96 (0.87–1.06)	0.96 (0.87–1.05)	**1.20 (1.10–1.32)**	1.01 (0.82–1.24)	0.99 (0.79–1.25)	1.19 (0.76–1.87)
Conduct disorder	0.98 (0.90–1.05)	0.97 (0.90–1.05)	**1.19 (1.12–1.27)**	0.93 (0.84–1.02)	0.91 (0.83–1.01)	**1.19 (1.09–1.30)**	1.07 (0.87–1.30)	1.06 (0.85–1.32)	1.24 (0.80–1.93)
Anxiety disorders	***0.74 (0.68–0.80)***	***0.71 (0.66–0.77)***	***0.90 (0.84–0.96)***	***0.67 (0.61–0.74)***	***0.66 (0.60–1.73)***	***0.83 (0.75–0.91)***	***0.54 (0.44–0.66)***	***0.53 (0.42–0.66)***	***0.63 (0.41–0.99)***
Post-traumatic and other stress-related disorders	***0.88 (0.81–0.95)***	***0.86 (0.80–0.93)***	1.04 (0.97–1.10)	***0.81 (0.74–0.89)***	***0.80 (0.72–0.88)***	0.99 (0.90–1.08)	***0.81 (0.66–0.99)***	0.81 (0.65–1.02)	0.87 (0.56–1.36)
Eating disorders	0.98 (0.91–1.06)	0.97 (0.90–1.05)	**1.14 (1.07–1.22)**	0.93 (0.85–1.03)	0.92 (0.84–1.10)	**1.16 (1.06–1.27)**	1.10 (0.90–1.35)	1.10 (0.88–1.38)	1.16 (0.74–1.82)
Depression and other mood disorders	***0.79 (0.73–0.85)***	***0.77 (0.71–0.83)***	0.94 (0.88–1.01)	***0.73 (0.66–0.80)***	***0.71 (0.65–0.78)***	***0.90 (0.82–0.99)***	***0.65 (0.53–0.80)***	***0.65 (0.52–0.81)***	0.71 (0.46–1.12)
Bipolar disorders	0.94 (0.87–1.02)	0.93 (0.86–1.01)	**1.13 (1.06–1.21)**	0.89 (0.81–0.99)	***0.88 (0.80–0.97)***	**1.13 (1.03–1.23)**	0.90 (0.73–1.10)	0.91 (0.72–1.14)	0.95 (0.61–1.49)
Schizophrenia and other psychotic disorders	0.96 (0.89–1.04)	0.95 (0.88–1.02)	**1.19 (1.12–1.27)**	0.92 (0.84–1.02)	0.91 (0.82–1.00)	**1.20 (1.09–1.31)**	0.87 (0.71–1.06)	0.84 (0.67–1.06)	1.06 (0.68–1.66)
Substance use disorders	***0.83 (0.77–0.89)***	***0.80 (0.74–0.87)***	**1.08 (1.01–1.15)**	***0.79 (0.72–0.87)***	***0.77 (0.70–0.85)***	1.07 (0.97–1.17)	***0.62 (0.51–0.75)***	***0.61 (0.49–0.76)***	0.69 (0.44–1.08)
Dissocial personality disorder	0.96 (0.89–1.04)	0.96 (0.89–1.03)	**1.20 (1.12–1.28)**	0.92 (0.83–1.01)	***0.90 (0.82–0.99)***	**1.19 (1.09–1.31)**	0.98 (0.80–1.20)	0.96 (0.77–1.21)	1.20 (0.77–1.86)

Starting from the second row and for the rest of the table, each row reports the results from the model with additional adjustment for a corresponding comorbid psychiatric disorder. Statistically significant hazard ratios are highlighted in bold

*CI* confidence interval, *HR* hazard ratio

^a^Adjusted for sex, birth year, interaction term (OCD × sex), and for a corresponding comorbid psychiatric disorder

^b^Adjusted for birth year and for a corresponding comorbid psychiatric disorder

^c^Adjusted for sex, birth year, and for a corresponding comorbid psychiatric disorder (interaction term is not included because sex does not modify an association between OCD and convictions)

**Fig. 2:**
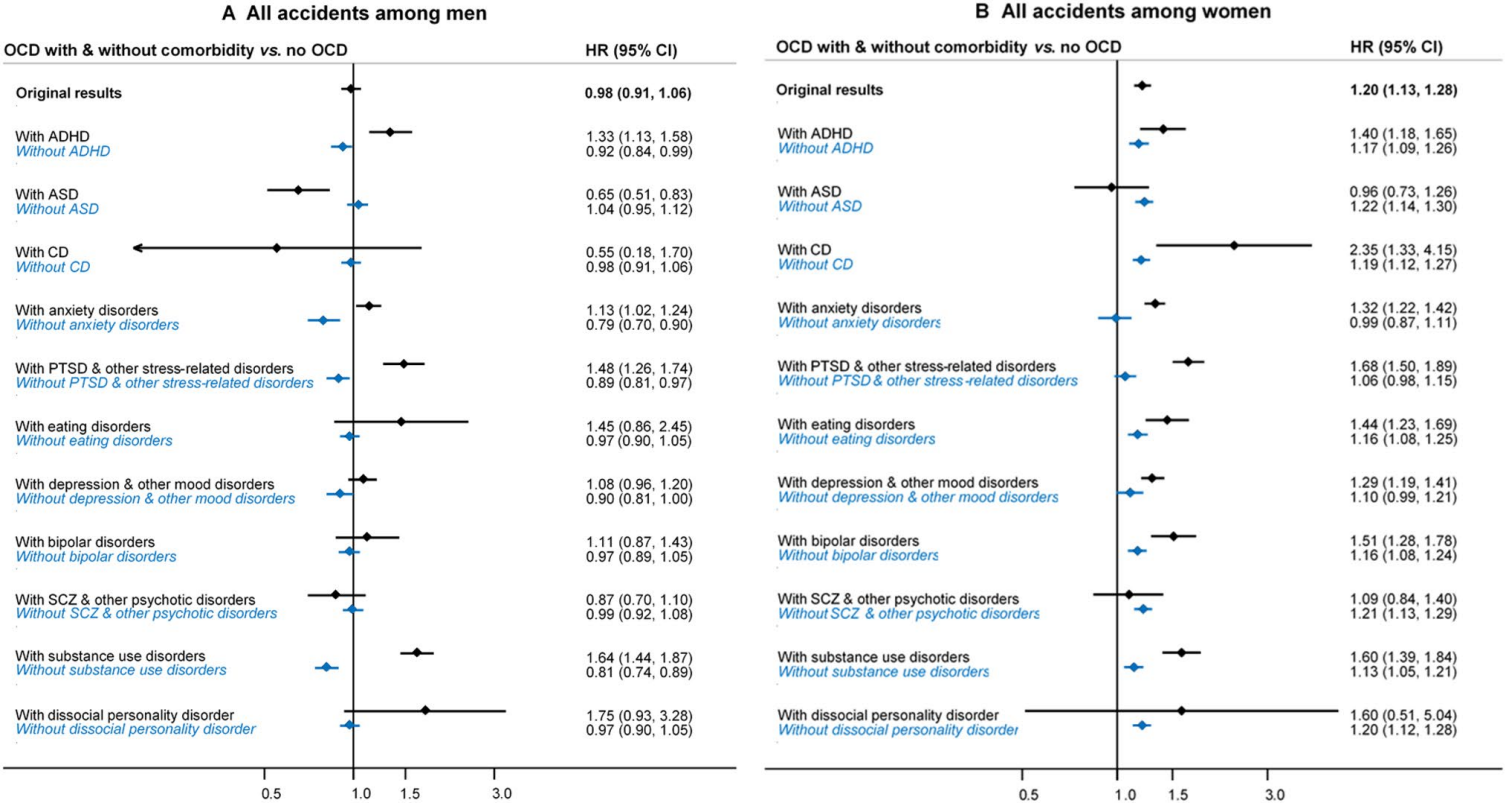
Hazard ratios and corresponding 95% confidence intervals for the risk of injuries and death due to transport accidents in men (panel A) and women (panel B) with obsessive-compulsive disorder with and without psychiatric comorbidities (one disorder group at the time), compared to their unaffected counterparts from the general population. *ADHD* attention-deficit/hyperactivity disorder, *ASD* autism spectrum disorders, *CD* conduct disorder, *CI* confidence interval, *HR* hazard ratio, *PTSD* post-traumatic stress disorders, *SCZ* schizophrenia

These findings were supported by the results of a series of post-hoc analyses where individuals with OCD were split by their comorbidity status (separately for each group of comorbid disorders) and compared to OCD-unaffected individuals (Fig. 2, Supplementary Table 3). In general, the co-occurrence of OCD with the above-mentioned comorbidities resulted in significant increase in the risk of the outcomes of interest. This was particularly obvious for comorbid ADHD, post-traumatic and other stress-related disorders, eating disorders, bipolar disorder, and substance use disorders. By contrast, in the absence of such comorbidities, individuals with OCD had either similar or even lower risks of these outcomes than general population individuals. Interestingly, comorbid OCD and ASD was associated with a *reduced* risk of some of the outcomes of interest.

## Discussion

Using the Swedish population-based registers, we formally investigated whether OCD is associated with an increased risk of serious transport accidents, defined as accidents requiring a specialist visit or resulting in death, and criminal convictions due to traffic offences in a large nationwide cohort. The main findings were that women, but not men, with OCD had a small (approximately 20%) but statistically significantly increased risk of serious transport accidents, compared to their unaffected counterparts from the general population. Neither women nor men with OCD had a significantly different risk of convictions related to traffic offenses (i.e., reckless driving, hit-and-run offenses, causing death or injury by driving, and moving violations). In a sub-cohort of individuals followed from age 18, the results followed a similar pattern but some of the associations (i.e., total sample, men) were inverted, indicating that individuals with OCD may have a lower risk of serious transport accidents and motor vehicle accidents. However, this sub-cohort was also younger, which means that the follow-up period may have been too short to detect an effect. Analyses of the sibling cohort, which provide a stricter control of unmeasured familial confounding, revealed no statistically significant associations between OCD and any of the outcomes of interest. Finally, when psychiatric comorbidities were systematically adjusted for, the initial results were considerably attenuated, in particular when ADHD, anxiety disorders, post-traumatic and other stress-related disorders, depression and other mood disorders, and substance use disorders were controlled for. This resulted in non-significant or inverse associations, again suggesting that the risks in individuals with OCD may be comparable or even lower than that among the general population.

Additional post-hoc analyses confirmed and extended the results of the planned analyses. Specifically, when the OCD cohort was split according to comorbidity status (one comorbidity group at a time), it was evident that certain comorbidities significantly increased the risk of serious transport accidents and criminal offences. In line with our initial hypothesis, comorbid ADHD increased the risk of all outcomes in both men and women with OCD, with HRs ranging from 1.32 to 2.72. In Tourette syndrome, a closely related disorder, ADHD comorbidity was also the single most important contributor to the increased risk of serious transport accidents in that patient group [26]. In general, these findings are in line with the known association between ADHD and traffic accidents [6, 7]. By contrast, for individuals with OCD who did not have comorbid ADHD, the results were much weaker: either marginally significant (only for women), non-significant or even inverse (for example, men with OCD and no ADHD had a 17% lower risk of motor vehicle accidents than men from the general population).

A number of other comorbidities also had a deleterious impact on the outcomes of interest, in particular anxiety disorders, stress related disorders, substance use disorders, and dissocial personality disorder. For example, individuals with OCD who had comorbid substance use disorders had significantly higher risks of all three outcomes of interest, with HRs ranging from 1.50 to 2.95. Again, individuals without such comorbidity had either negligible risks (only women with OCD) or even significantly lower risks than the population controls. Perhaps unsurprisingly, individuals with comorbid dissocial personality disorder had the highest risk of driving-related criminal convictions, though the confidence intervals were broad due to a small number of cases with such comorbidity. Interestingly, we found that individuals with OCD and comorbid ASD had a significantly *reduced* risk of serious transport accidents. Given the difficulties with driving experienced by autistic individuals [27, 28], we speculate that individuals with a diagnosis of ASD may be less likely to drive regularly or to obtain a driver’s license [29].

It is important to note that the proportion of persons with OCD involved in a serious transport accidents and related criminal offences was small, even in comorbid cases. For example, only 6.86% of all OCD patients (regardless of comorbidity status) were involved in serious transport accidents compared to 5.63% from the general population. The corresponding figures for motor vehicle accidents were even smaller (3.84% and 3.12%, respectively) and driving-related criminal convictions were very rare events (0.42% and 0.36%, respectively). Collectively, these results indicate that the risk of serious transport accidents in OCD is negligible. The marginally increased risk observed in women is likely to reflect the relatively lower rate of these outcomes in the general female population, rather than absolute differences in risk between women and men with OCD.

### Implications for clinical practice and vehicle licensing agencies

First and foremost, clinicians are advised to share our results with their patients who struggle with ‘hit-and-run’ OCD, as many of these patients perceive themselves as constituting a serious danger to others. While this knowledge will not substitute the need for patients to practice exposure and response prevention tasks or carry out behavioural experiments during their cognitive behaviour therapy, having access to objective data from a nationwide study may be a powerful tool.

Second, managing of comorbidities that may potentially increase the risk of serious transport accidents in this group is important. In particular, there is good evidence that adequate management of comorbid ADHD is associated with a significantly reduced risk of severe road traffic injuries and deaths [6, 30]. It is currently unknown if successful treatment of other comorbid conditions will lead to further risk reduction in comorbid OCD cases.

From a licensing authority perspective, our data suggest that the majority of individuals with OCD will be able to drive safely, and therefore licensing agencies should not necessarily equate a diagnosis of OCD with an increased risk of serious transport accidents, even if some patients may perceive themselves as being a danger to others. Instead, any licensing decisions should be made on an individual basis and primarily focus on comorbid conditions, rather than the diagnosis of OCD itself.

### Limitations

The results should be interpreted in light of some limitations. First, because the registers do not contain OCD severity information, we could not examine the impact of this potentially important variable on the risks. Neither did we have information on the patients’ individual symptoms, which means that we could not examine the risks of the outcomes of interest in individuals with specific fears of driving. Second, our study focused on outcomes requiring hospital/specialized outpatient clinic visits or deaths attributable to transport accidents. Thus, we had no access to information on milder accidents not requiring such care (e.g., individuals treated at primary care). Third, based on the available ICD codes for accidents, we cannot know whether the person involved in the accident was the driver or a passenger. However, we made the assumption that the probability of being involved in a transport accident as a passenger should not be higher for individuals with OCD than for individuals from the general population. Fourth, to our knowledge, there are no published data regarding the probability that individuals with OCD will obtain a drivers’ license or limit their driving; if individuals with OCD were less likely to obtain a license or to actually drive, compared to individuals from the general population, that would potentially result in an underestimation of the true risk of transport accidents in this patient group. Finally, the results of the sibling analyses need to be interpreted in light of the known limitations of such designs [31].

## Supplementary Information

Below is the link to the electronic supplementary material.

Supplementary file1 (DOCX 42 kb)

## Declarations

### Data availability

The data that support the findings of this study are not publicly available because of the Swedish Secrecy Act. Researchers may apply for access through the Swedish Research Ethics Boards (www.etikprovningsmyndigheten.se) and from the primary data owners: Statistics Sweden (www.scb.se) and the National Board of Health and Welfare (www.socialstyrelsen.se), in accordance with Swedish law.

### Code availability

Stata code used in this study can be made available to researchers upon request to the correspondent author.
