# Supplementary material for: Hit-and-run: a Swedish nationwide cohort study of serious transport accidents and convictions due to traffic offenses in obsessive–compulsive disorder
Source: Soc Psychiatry Psychiatr Epidemiol. 2021 Nov 15;57(9):1817–27. doi: 10.1007/s00127-021-02182-x (PMC9375758; doi:10.1007/s00127-021-02182-x)
Supplement: Supplementary file 1 — Supplementary file1 (DOCX 42 kb) [file 127_2021_2182_MOESM1_ESM.docx]

**SUPPLEMENTARY MATERIAL**

**Supplementary Table 1.** List of Swedish International Classification of Diseases, 10th edition (ICD-10) codes to collect records of psychiatric disorders from the National Patient Register. Age constraints are applied to avoid risk of misclassification.

|  | **Swedish ICD-10 codes** | **Minimal age (years)** |
| --- | --- | --- |
| Autism spectrum disorders | F84.0, .1, .3, .5, .8, .9 | 1 |
| Attention-deficit/hyperactivity disorder | F90 (plus individuals with the following dispensed drugs: N06BA04, N06BA01, N06BA02, N06BA09, or N06BA12)^a^ | 3 |
| Conduct disorder | F91 | 3 |
| Anxiety disorders | F40, F41 | 6 |
| Posttraumatic and other stress-related disorders | F43 | 6 |
| Eating disorders | F50.0-F50.3, F50.9 | 8 |
| Depression and other mood disorders | F32, F33, F34 (minus F34.0), F38, F39 | 6 |
| Bipolar disorders | F25.0, F30, F31, F34.0 | 10 |
| Schizophrenia and other psychotic disorders | F20, F21, F22, F23, F24, F25 (minus F25.0), F28, F29 | 10 |
| Substance use disorders | F10-F16 and F18-19 | 10 |
| Dissocial personality disorder | F60.2 | 18 |

^a^ As recommended in previous epidemiological studies using the Swedish national registers, the diagnosis of attention-deficit/hyperactivity disorder was ascertained using both International Classification of Diseases (ICD) codes in the National Patient Register and medications usually prescribed for the treatment of the disorder, using Anatomical Therapeutic Chemical (ATC) codes, in the Prescribed Drug Register. See Chen et al. (2016). Familial aggregation of attention-deficit/hyperactivity disorder. *The Journal of Child Psychology and Psychiatry, 58*(3), 231-239.

**Supplementary Table 2**. Hazard ratios and corresponding 95% confidence intervals for the risk of injury or death due to transport accidents, motor vehicle accidents, and convictions due to traffic offenses among individuals with obsessive-compulsive disorder, compared to unaffected individuals from the general population, in individuals followed from age 18 years.

|  | **Individuals with OCD**  **(n=11,734)** | | **Unaffected individuals**  **(n=1,541,189)** | |  |
| --- | --- | --- | --- | --- | --- |
| ***Outcomes*** | **n** | **%** | **n** | **%** | ***HR (*95*% CI)*** |

| **All serious transport accidents** | 732 | 6.24 | 98,297 | 6.38 | ***0.75 (0.66-0.85)*^‡a^** |
| --- | --- | --- | --- | --- | --- |
| Men | 255 | 5.50 | 55,763 | 7.04 | ***0.74 (0.66-0.84)*^‡b^** |
| Women | 477 | 6.72 | 42,534 | 5.68 | **1.12 (1.03-1.23)^§b^** |
| **Motor vehicle accidents** | 436 | 3.72 | 63,589 | 4.13 | ***0.71 (0.61-0.83)* ^‡a^** |
| Men | 177 | 3.82 | 40,873 | 5.16 | ***0.71 (0.61-0.82)***^‡b^ |
| Women | 259 | 3.65 | 22,716 | 3.03 | **1.14 (1.01-1.29)**^§b^ |
| **Convictions due to traffic offenses** | 32 | 0.27 | 6,969 | 0.45 | 0.71 (0.50-1.01)^c^ |
| Men | 27 | 0.58 | 6,282 | 0.79 | 0.71 (0.49-1.04)^b^ |
| Women | 5 | 0.07 | 687 | 0.09 | 0.72 (0.30-1.74)^b^ |

*Note:* Statistically significant hazard ratios are highlighted in bold. Mean length of follow-up and SD for analyses of outcomes: all transport accidents 8.04 years (SD=4.62), motor vehicle accidents 8.16 years (SD=4.61), convictions due to traffic offences 8.35 years (SD=4.59).

a Adjusted for sex, birth year and interaction term (OCD × sex)

b Adjusted for birth year

c Adjusted for sex and birth year (interaction term is not included since sex does not modify an association between OCD and convictions)

§ p-value <0.05, † p-value <0.01, ‡ p-value <0.001.

*Abbreviations:* CI confidence interval, HR hazard ratio, OCD obsessive-compulsive disorder, SD standard deviation.

**Supplementary Table 3**. Hazard ratios and corresponding 95% confidence intervals for the risk of injuries and death due to transport accidents, motor vehicle accidents, and convictions due the traffic offenses in individuals with obsessive-compulsive disorder with and without psychiatric comorbidities (one disorder group at the time), compared to unaffected individuals from the general population.

|  | **HR (95% CI)** | | | | | | | | |
| --- | --- | --- | --- | --- | --- | --- | --- | --- | --- |
|  | **All transport accidents** | | | **Motor vehicle accidents** | | | **Convictions due to traffic offenses** | | |
|  | **All^a^** | **Men^b^** | **Women^b^** | **All^a^** | **Men^b^** | **Women^b^** | **All^c^** | **Men^b^** | **Women^b^** |
| **Original results** | 0.98 (0.91-1.06) | 0.98 (0.91-1.06) | **1.20 (1.13-1.28)** | 0.94 (0.85-1.03) | 0.92 (0.84-1.02) | **1.20 (1.09-1.31)** | 1.11 (0.91-1.35) | 1.10 (0.88-1.38) | 1.26 (0.81-1.96) |
| No OCD | 1.0 | 1.0 | 1.0 | 1.0 | 1.0 | 1.0 | 1.0 | 1.0 | 1.0 |
| OCD with attention-deficit/ hyperactivity disorder | **1.34 (1.13-1.59)** | **1.33 (1.13-1.58)** | **1.40 (1.18-1.65)** | **1.45 (1.19-1.76)** | **1.42 (1.17-1.72)** | **1.32 (1.04-1.67)** | **2.72 (1.59-3.25)** | **2.26 (1.52-3.35)** | **2.59 (1.08-6.23)** |
| OCD without attention-deficit/ hyperactivity disorder | 0.92 (0.85-1.00) | ***0.92 (0.84-0.99)*** | **1.17 (1.09-1.26)** | ***0.84 (0.75-0.94)*** | ***0.83 (0.74-0.93)*** | **1.18 (1.07-1.30)** | 0.90 (0.71-1.15) | 0.88 (0.67-1.16) | 1.08 (0.65-1.79) |
| OCD with autism spectrum disorders | ***0.66 (0.52-0.84)*** | ***0.65 (0.51-0.83)*** | 0.96 (0.73-1.26) | ***0.59 (0.44-0.80)*** | ***0.58 (0.43-0.79)*** | 0.83 (0.55-1.25) | 0.69 (0.35-1.38) | 0.75 (0.37-1.50) | na |
| OCD without autism spectrum disorders | 1.04 (0.96-1.13) | 1.04 (0.95-1.12) | **1.22 (1.14-1.30)** | 1.00 (0.90-1.11) | 0.99 (0.89-1.09) | **1.23 (1.12-1.35)** | 1.17 (0.95-1.44) | 1.16 (0.92-1.48) | 1.35 (0.87-2.09) |
| OCD with conduct disorder | 0.55 (0.18-1.68) | 0.55 (0.18-1.70) | **2.35 (1.33-4.15)** | 0.52 (0.13-2.06) | 0.51 (0.13-2.03) | 2.19 (0.98-4.87) | 2.99 (0.75-12.03) | 3.55 (0.88-14.30) | na |
| OCD without conduct disorder | 0.99 (0.92-1.07) | 0.98 (0.91-1.06) | **1.19 (1.12-1.27)** | 0.94 (0.85-1.04) | 0.93 (0.84-1.02) | **1.19 (1.09-1.31)** | 1.09 (0.89-1.34) | 1.08 (0.86-1.36) | 1.27 (0.82-1.97) |
| OCD with anxiety disorders | **1.14 (1.03-1.25)** | **1.13 (1.02-1.24)** | **1.32 (1.22-1.42)** | 1.09 (0.96-1.23) | 1.07 (0.95-1.21) | **1.28 (1.15-1.43)** | **1.30 (1.02-1.66)** | **1.34 (1.02-1.76)** | 1.35 (0.80-2.29) |
| OCD without anxiety disorders | ***0.80(0.70-0.91)*** | ***0.79 (0.70-0.90)*** | 0.99 (0.87-1.11) | ***0.75 (0.64-0.88)*** | ***0.74 (0.63-0.87)*** | 1.04 (0.88-1.22) | 0.84 (0.59-1.19) | 0.80 (0.54-1.19) | 1.09 (0.49-2.43) |
| OCD with post-traumatic and other stress-related disorders | **1.49 (1.27-1.75)** | **1.48 (1.26-1.74)** | **1.68 (1.50-1.89)** | **1.55 (1.28-1.88)** | **1.53 (1.26-1.85)** | **1.79 (1.53-2.09)** | **1.74 (1.18-2.56)** | 1.49 (0.91-2.44) | **2.74 (1.47-5.10)** |
| OCD without post-traumatic and other stress-related disorders | ***0.89 (0.82-0.98)*** | ***0.89 (0.81-0.97)*** | 1.06 (0.98-1.15) | ***0.83 (0.74-0.93)*** | ***0.82 (0.73-0.91)*** | 1.02 (0.92-1.15) | 0.98 (0.77-1.23) | 1.03 (0.80-1.32) | 0.82 (0.44-1.53) |
| OCD with eating disorders | 1.45 (0.86-2.46) | 1.45 (0.86-2.45) | **1.44 (1.23-1.69)** | 1.03 (0.49-2.18) | 1.01 (0.48-2.14) | 1.17 (0.91-1.49) | 0.78 (0.25-2.44) | na | 1.51 (0.49-4.69) |
| OCD without eating disorders | 0.98 (0.82-1.06) | 0.97 (0.90-1.05) | **1.16 (1.08-1.25)** | 0.94 (0.85-1.03) | 0.92 (0.84-1.02) | **1.20 (1.09-1.33)** | 1.12 (0.92-1.37) | 1.12 (0.89-1.40) | 1.23 (0.76-1.98) |
| OCD with depression and other mood disorders | 1.09 (0.97-1.21) | 1.08 (0.96-1.20) | **1.29 (1.19-1.41)** | 1.05 (0.92-1.21) | 1.04 (0.90-1.19) | **1.22 (1.07-1.38)** | 1.07 (0.79-1.44) | 1.08 (0.77-1.52) | 1.18 (0.64-2.21) |
| OCD without depression and other mood disorders | 0.91 (0.82-1.01) | 0.90 (0.81-1.00) | 1.10 (0.99-1.21) | ***0.85 (0.74-0.97)*** | ***0.84 (0.73-0.96)*** | **1.18 (1.03-1.35)** | 1.14 (0.87-1.49) | 1.12 (0.83-1.50) | 1.35 (0.73-2.52) |
| OCD with bipolar disorders | 1.12 (0.87-1.44) | 1.11 (0.87-1.43) | **1.51 (1.28-1.78)** | 0.88 (0.62-1.24) | 0.87 (0.61-1.23) | **1.45 (1.14-1.84)** | 1.63 (0.95-2.80) | 1.76 (0.95-3.26) | 1.52 (0.49-4.73) |
| OCD without bipolar disorders | 0.97 (0.90-1.05) | 0.97 (0.89-1.05) | **1.16 (1.08-1.24)** | 0.94 (0.85-1.04) | 0.93 (0.84-1.03) | **1.16 (1.05-1.28)** | 1.06 (0.85-1.31) | 1.04 (0.82-1.33) | 1.23 (0.76-1.98) |
| OCD with schizophrenia and other psychotic disorders | 0.88 (0.70-1.11) | 0.87 (0.70-1.10) | 1.09 (0.84-1.40) | ***0.66 (0.48-0.91)*** | ***0.65 (0.47-0.90)*** | 1.05 (0.73-1.51) | 1.52 (0.91-2.52) | 1.61 (0.95-2.72) | 0.81 (0.11-5.77) |
| OCD without schizophrenia and other psychotic disorders | 1.00 (0.92-1.08) | 0.99 (0.92-1.08) | **1.21 (1.13-1.29)** | 0.98 (0.88-1.08) | 0.96 (0.87-1.07) | **1.21 (1.10-1.33)** | 1.06 (0.85-1.31) | 1.03 (0.80-1.32) | 1.30 (0.83-2.05) |
| OCD with substance use disorders | **1.66 (1.45-1.89)** | **1.64 (1.44-1.87)** | **1.60 (1.39-1.84)** | **1.64 (1.39-1.93)** | **1.62 (1.38-1.90)** | **1.50 (1.23-1.84)** | **2.87 (2.17-3.80)** | **2.95 (2.18-3.99)** | **2.76 (1.31-5.81)** |
| OCD without substance use disorders | ***0.82 (0.74-0.90)*** | ***0.81 (0.74-0.89)*** | **1.13 (1.05-1.21)** | ***0.76 (0.68-0.86)*** | ***0.75 (0.67-0.85)*** | **1.14 (1.03-1.26)** | ***0.68 (0.51-0.90)*** | ***0.63 (0.45-0.88)*** | 0.98 (0.57-1.69) |
| OCD with dissocial disorder | 1.77 (0.94-3.31) | 1.75 (0.93-3.28) | 1.60 (0.51-5.04) | **2.32 (1.20-4.47)** | **2.28 (1.18-4.41)** | 1.05 (0.14-7.56) | **9.67 (4.31-21.69)** | **8.49 (3.51-20.56)** | **25.22 (3.63-174.93)** |
| OCD without dissocial disorder | 0.98 (0.91-1.06) | 0.97 (0.90-1.05) | **1.20 (1.12-1.28)** | 0.92 (0.84-1.02) | 0.91 (0.83-1.00) | **1.20 (1.09-1.31)** | 1.05 (0.85-1.29) | 1.04 (0.82-1.31) | 1.20 (0.77-1.89) |

*Note:* Statistically significant hazard ratios are highlighted in bold.

a Adjusted for sex, birth year and interaction term (OCD × sex)

b Adjusted for birth year

c Adjusted for sex and birth year (interaction term is not included since sex does not modify an association between OCD and convictions)
